# Comparison of machine learning and conventional criteria in detecting left ventricular hypertrophy and prognosis with electrocardiography

**DOI:** 10.1093/ehjdh/ztaf003

**Published:** 2025-02-11

**Authors:** Jui-Tzu Huang, Chih-Hsueh Tseng, Wei-Ming Huang, Wen-Chung Yu, Hao-Min Cheng, Hsi-Lu Chao, Chern-En Chiang, Chen-Huan Chen, Albert C Yang, Shih-Hsien Sung

**Affiliations:** Department of Medicine, Taipei Veterans General Hospital, No. 201, Sec. 2, Shipai Rd., Beitou District, Taipei 112, Taiwan; Department of Internal Medicine, National Yang Ming Chiao Tung University, No. 155, Sec. 2, Linong Street, Beitou District, Taipei 112, Taiwan; Department of Medicine, Taipei Veterans General Hospital, No. 201, Sec. 2, Shipai Rd., Beitou District, Taipei 112, Taiwan; Department of Internal Medicine, National Yang Ming Chiao Tung University, No. 155, Sec. 2, Linong Street, Beitou District, Taipei 112, Taiwan; Department of Medicine, Taipei Veterans General Hospital, No. 201, Sec. 2, Shipai Rd., Beitou District, Taipei 112, Taiwan; Department of Internal Medicine, National Yang Ming Chiao Tung University, No. 155, Sec. 2, Linong Street, Beitou District, Taipei 112, Taiwan; Department of Medicine, Kinmen Hospital, Ministry of Health and Welfare, No. 2, Fuxing Rd., Jinhu Township, Kinmen County 891, Taiwan; Department of Medicine, Taipei Veterans General Hospital, No. 201, Sec. 2, Shipai Rd., Beitou District, Taipei 112, Taiwan; Department of Internal Medicine, National Yang Ming Chiao Tung University, No. 155, Sec. 2, Linong Street, Beitou District, Taipei 112, Taiwan; Department of Internal Medicine, National Yang Ming Chiao Tung University, No. 155, Sec. 2, Linong Street, Beitou District, Taipei 112, Taiwan; Department of Medical Education, Taipei Veterans General Hospital, Taipei, Taiwan; Cardiovascular Research Center, National Yang Ming Chiao Tung University, No. 155, Sec. 2, Linong Street, Beitou District, Taipei 112, Taiwan; Department of Computer Science, National Yang Ming Chiao Tung University, No. 155, Sec. 2, Linong Street, Beitou District, Taipei 112, Taiwan; Department of Internal Medicine, National Yang Ming Chiao Tung University, No. 155, Sec. 2, Linong Street, Beitou District, Taipei 112, Taiwan; Cardiovascular Research Center, National Yang Ming Chiao Tung University, No. 155, Sec. 2, Linong Street, Beitou District, Taipei 112, Taiwan; General Clinical Research Center, Taipei Veterans General Hospital, No. 201, Sec. 2, Shipai Rd., Beitou District, Taipei 112, Taiwan; Department of Medicine, Taipei Veterans General Hospital, No. 201, Sec. 2, Shipai Rd., Beitou District, Taipei 112, Taiwan; Department of Internal Medicine, National Yang Ming Chiao Tung University, No. 155, Sec. 2, Linong Street, Beitou District, Taipei 112, Taiwan; Cardiovascular Research Center, National Yang Ming Chiao Tung University, No. 155, Sec. 2, Linong Street, Beitou District, Taipei 112, Taiwan; Department of Medicine, Taipei Veterans General Hospital, No. 201, Sec. 2, Shipai Rd., Beitou District, Taipei 112, Taiwan; Cardiovascular Research Center, National Yang Ming Chiao Tung University, No. 155, Sec. 2, Linong Street, Beitou District, Taipei 112, Taiwan; Department of Medicine, Taipei Veterans General Hospital, No. 201, Sec. 2, Shipai Rd., Beitou District, Taipei 112, Taiwan; Department of Internal Medicine, National Yang Ming Chiao Tung University, No. 155, Sec. 2, Linong Street, Beitou District, Taipei 112, Taiwan; Cardiovascular Research Center, National Yang Ming Chiao Tung University, No. 155, Sec. 2, Linong Street, Beitou District, Taipei 112, Taiwan; General Clinical Research Center, Taipei Veterans General Hospital, No. 201, Sec. 2, Shipai Rd., Beitou District, Taipei 112, Taiwan; Institute of Emergency and Critical Care Medicine, National Yang Ming Chiao Tung University, No. 155, Sec. 2, Linong Street, Beitou District, Taipei 112, Taiwan

**Keywords:** Artificial intelligence, Electrocardiogram, Left ventricular hypertrophy, Prognosis

## Abstract

**Aims:**

Left ventricular hypertrophy (LVH) is clinically important; current electrocardiography (ECG) diagnostic criteria are inadequate for early detection. This study aimed to develop an artificial intelligence (AI)-based algorithm to improve the accuracy and prognostic value of ECG criteria for LVH detection.

**Methods and results:**

A total of 42 016 patients (64.3 ± 16.5 years, 55.3% male) were enrolled. LV mass index was calculated from echocardiographic measurements. Left ventricular hypertrophy screening utilized ECG criteria, including Sokolow–Lyon, Cornell product, Cornell/strain index, Framingham criterion, and Peguero–Lo Presti. An AI algorithm using CatBoost was developed and validated (training dataset 80% and testing dataset 20%). F1 scores, reflecting the harmonic mean of precision and recall, were calculated. Mortality data were obtained through linkage with the National Death Registry. The CatBoost-based AI algorithm outperformed conventional ECG criteria in detecting LVH, achieving superior sensitivity, specificity, positive predictive value, F1 score, and area under curve. Significant features to predict LVH involved QRS and P-wave morphology. During a median follow-up duration of 10.1 years, 1655 deaths occurred in the testing dataset. Cox regression analyses showed that LVH identified by AI algorithm (hazard ratio and 95% confidence interval: 1.587, 1.309–1.924), Sokolow–Lyon (1.19, 1.038–1.365), Cornell product (1.301, 1.124–1.505), Cornell/strain index (1.306, 1.185–1.439), Framingham criterion (1.174, 1.062–1.298), and echocardiography-confirmed LVH (1.124, 1.019–1.239) were all significantly associated with mortality. Notably, AI-diagnosed LVH was more predictive of mortality than echocardiography-confirmed LVH.

**Conclusion:**

Artificial intelligence-based LVH diagnosis outperformed conventional ECG criteria and was a superior predictor of mortality compared to echocardiography-confirmed LVH.

## Graphical Abstract

**Graphical Abstract:**
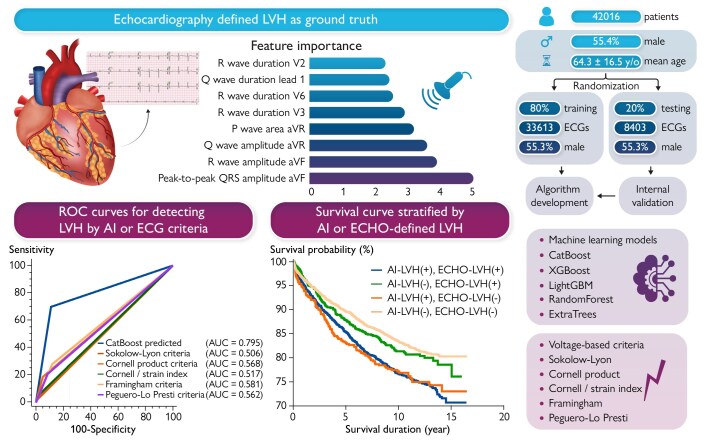

## Introduction

Left ventricular hypertrophy (LVH), implicating cardiac remodelling, is a well-known prognostic factor for cardiovascular disease, adverse events, and long-term mortality.^1,2^ Early detection of LVH could be of great clinical significance, as timely intervention can improve patient outcomes.^3–5^ Although echocardiogram is the standard method for assessing LVH,^6^ medical accessibility limits its use in screening. Electrocardiogram (ECG) has been a cost-effective tool to predict the probability of LVH for decades.^7^ Even though there are numerous voltage criteria for diagnosing LVH, unsatisfactory accuracy with low sensitivity and poor positive predictive values have been reported.^8–10^ In addition, racial discrepancies have yet to be evaluated. Given that Asians tend to have smaller body sizes and thinner chest walls than Caucasians^11–13^, the ECG criteria should be modified for broader clinical applications.^11^

Nowadays, the available diagnostic ECG-LVH criteria have largely involved QRS morphology.^14^ However, electrical remodelling of the QRS complex with diffuse or regionally slowed conduction velocity could be also found in non-hypertrophic left ventricles, which explains the discrepancies observed between anatomical LVH and ECG characteristics.^15,16^ Computer-assisted and digital interpretation of ECGs has become integrally in clinical evaluation and serves as an adjunct to physician interpretation over recent decades. Machine learning can be more efficient in extracting ECG features than manual observations in refining ECG-LVH criteria.^17–19^

In this study, we aimed to compare the diagnostic accuracy and prognostic impacts of machine learning with traditional ECG criteria for LVH in an Asian population. We further identified specific ECG features to optimize the ECG-LVH diagnostic criteria for clinical applications.

## Methods

### Study population

**Figure 1:**
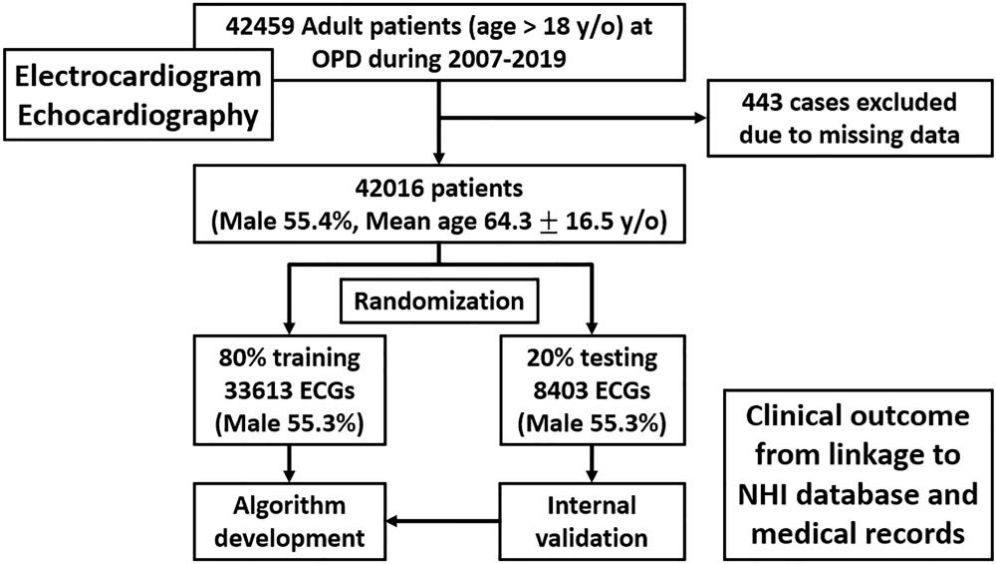
Flow chart of the study population.

Ambulatory subjects who received both ECG and echocardiography within a 2-week interval at outpatient clinics from January 2007 to December 2019 were included in this study. *Figure 1* demonstrated the flow chart of the study population. Patients hospitalized for acute diseases within a month were excluded. Anthropometric data and comorbidities were collected from the medical HIS system of Taipei Veteran General Hospital.

### Ethical approvals

The investigation conformed to the principles outlined in the Declaration of Helsinki. The institutional review board of Taipei Veterans General Hospital waived the informed consent and approved this study.

### Data collection and processing

All 12-lead ECGs were performed using a 25 mm/s velocity and 10 mm/mV calibration (Philips Healthcare), which were then decomposed using the Philips DXL algorithm to obtain 527 parameters per recording, including heart rate, quantified analysis, and numerical summaries of amplitude, duration, area, and shape for every P-wave, QRS complex, ST segment, and T-wave in each lead (see Supplementary material online, *Table S1*). Traditional ECG-LVH criteria were used to evaluate LVH, including Sokolow–Lyon (SV1 + RV5 or RV6 ≥ 3.5 mV), Cornell voltage product [(S V3 + RaVL) × QRS-duration for men and (SV3 + RaVL + 0.6) × QRS-duration for women],^20^ Cornell strain index (typical strain pattern such as down-sloping ST-segment depression of at least 50 mV and asymmetrical T-wave inversion in any of the following leads: I, II, aVL, V2, V3, V4, V5, or V6, or SV3 + RaVL > 2.4 mV in men, >2.0 mV in women), Framingham criterion [at least one of the following: RaVL > 1.1 mV, SIII + RI ≥ 2.5 mV, SV1 or SV2 + RV5 or RV6 ≥ 3.5 mV, SV1 or SV2 (right precordial leads) ≥ 2.5 mV, RV5 or RV6 (left precordial leads) ≥ 2.5 mV, and typical strain pattern in V5 or V6],^21^ and Peguero–Lo Presti (deepest S wave in any single lead SD + SV4 > 2.3 mV for women and >2.8 mV for men).^22^

Standard echocardiographic studies were conducted, conforming to the guidance of the American Society of Echocardiography.^23^ Left ventricular internal dimensions (LVIDd), interventricular septal thickness (IVSd), posterior wall at diastole (PWd), and left ventricular ejection fraction (LVEF) were recorded accordingly.^13^ Relative wall thickness and right ventricular systolic pressure (RVSP) were calculated. Left ventricular mass (LVM) index was calculated using the Teichholz formula with M-mode data and adjusted for body height. Left ventricular hypertrophy was defined as LVM index > 47 g/m^2^ in females and >51 g/m^2^ in males.^24,25^

### Development of machine learning algorithm

We randomly separated our data into a training group (80%) for algorithm development and testing group (20%) for internal validation. We compared several machine learning models including CatBoost, XGBoost, LightGBM, RandomForest, and ExtraTrees. CatBoost, an algorithm for gradient boosting on decision trees, was selected as our machine learning approach based on its accuracy in detecting LVH (see Supplementary material online, *Table S2*). The feature selection method included in the CatBoost package was also implemented, and features with greater importance were plotted as bar charts.

### Patient follow-up and outcome ascertainment

Dates and causes of mortality for the study cohort were obtained by linking our database with the National Death Registry through a unique personal identification number given to every Taiwanese citizen.

### Statistical analysis

Data are expressed as means ± standard deviation for continuous variables and percentages for categorical variables. Between-group comparisons were conducted using Student’s *t*-test and χ^2^ tests. Sensitivity, specificity, positive predictive value, negative predictive value, and F1 scores^26^ (harmonic mean of the precision and recall) were assessed for model performance evaluation. Receiver operating characteristic curve analyses were conducted for each ECG-LVH criterion, and the area under curve (AUC) were calculated and compared by DeLong’s test. The predictive value of echocardiography-defined LVH (ECHO-LVH) and electrocardiography-defined LVH (ECG-LVH) for all-cause mortality was assessed using Cox proportional hazards regression survival analysis in the test group. Kaplan–Meier survival curve analyses, stratified by either echocardiogram- or AI-defined LVH, were performed. The independent prognostic values of ECHO-LVH and AI-LVH were assessed using multivariate Cox regression analysis, adjusting for age, gender, hypertension, diabetes, coronary artery disease (CAD), prior myocardial infarction (MI), atrial fibrillation (AF), and chronic obstructive pulmonary disease. Additionally, a forward stepwise Cox regression analysis was performed for AI-LVH and ECHO-LVH with fixed adjustments for the same variables to determine their relative predictive superiority. Net reclassification improvement (NRI) was used to evaluate the added predictive value of AI-LVH or ECHO-LVH beyond the multivariate model.^27–29^ Risk categories were defined using cut-off points of 9% (representing a 5-year mortality risk) and 20% (reflecting the overall population mortality risk) to classify low-, intermediate-, and high-risks. Subgroup analyses were performed to assess the consistency of the association between AI-LVH and all-cause mortality, stratified by age, gender, and comorbidities. All analyses were conducted using R Studio Team (2020) (PBC, Boston, MA, USA) and Python [Van Rossum, G., & Drake, F. L. (2009), Python 3 Reference Manual, Scotts Valley, CA: CreateSpace]. Values of two-tailed *P* < 0.05 were considered statistically significant.

## Results

### Baseline characteristics of the study population

A total of 42 016 cases were included in the analysis, and the baseline characteristics of the study population stratified by ECHO-LVH are listed in *Table 1*. Compared with the others, subjects with LVH were older, less likely to be male, and had more comorbidities, including hypertension, diabetes, CAD, prior MI, AF, and chronic obstructive pulmonary disease. LVIDd, IVSd, PWd, relative wall thickness, LVM, LVM index, and RVSP were greater, while LVEF was lower in subjects with LVH than those without.

**Table 1:** Baseline characteristics of study population

Variable	All (*n* = 42 016)	LVH (*n* = 16 100)	Non-LVH (*n* = 25 916)	*P* value
Age, years	64.3 ± 16.5	69.3 ± 14.0	61.2 ± 17.2	<0.001
Men, *n* (%)	23 243 (55.3)	8625 (53.6)	14 618 (56.4)	<0.001
Morbidities, *n* (%)				
Hypertension	16 106 (38.3)	8264 (51.3)	7842 (30.3)	<0.001
Diabetes mellitus	4949 (11.8)	2467 (15.3)	2482 (9.6)	<0.001
Coronary artery disease	8009 (19.1)	4092 (25.4)	3917 (15.1)	<0.001
Prior myocardial infarction	2749 (6.54)	1465 (9.10)	1284 (4.95)	<0.001
Atrial fibrillation	4791 (11.40)	2044 (12.69)	2747 (10.60)	<0.001
COPD	2191 (5.21)	1017 (6.32)	1174 (4.53)	<0.001
Echocardiographic characteristics				
LVIDd, mm	47.8 ± 8.3	52.3 ± 9.5	45.0 ± 5.9	<0.001
IVSd, mm	10.3 ± 2.4	11.6 ± 2.7	9.4 ± 1.7	<0.001
PWd, mm	10.1 ± 4.1	11.3 ± 6.2	9.3 ± 1.5	<0.001
LVEF, %	57.7 ± 9.0	55.6 ± 11.0	59.0 ± 7.2	<0.001
RVSP, mmHg	33.5 ± 13.4	36.8 ± 14.5	31.4 ± 12.3	<0.001
LVM, g	191.1 ± 20.0	271.4 ± 32.3	141.2 ± 36.8	<0.001
LVM index, g/m^2^	51.0 ± 5.3	74.1 ± 8.6	36.7 ± 7.9	<0.001

COPD, chronic obstructive pulmonary disease; LVIDd, left ventricular internal dimension at diastole; IVSd, interventricular septal thickness at diastole; PWd, posterior wall at diastole; LVEF, left ventricular ejection fraction; RVSP, right ventricular systolic pressure; LVM, left ventricular mass.

### Diagnostic performance of electrocardiography-left ventricular hypertrophy criteria

CatBoost-derived LVH showed the highest sensitivity, specificity, and positive predictive value, whereas Cornell product, Sokolow–Lyon, Cornell/strain index, and Peguero–Lo Presti provided better negative predictive values. The overall diagnostic performance, as evaluated by F1 scores, was greatest with CatBoost-defined LVH, followed by Framingham criteria, Peguero–Lo Presti, Cornell/strain index, Sokolow–Lyon, and Cornell product (*Table 2*). In addition, the CatBoost-based ECG algorithm achieved a higher AUC for detecting LVH compared to conventional ECG criteria. Across various subpopulations, the CatBoost-based ECG algorithm effectively predicted LVH, irrespective of age, gender, or comorbidities (*Table 3*).

**Table 2:** Diagnostic performance of electrocardiography-left ventricular hypertrophy criteria in the validation sample (*n* = 8403)

LVH criteria	Sensitivity, %	Specificity, %	PPV, %	NPV, %	F1 score, %	AUC
CatBoost predicted	80.9	84.2	73.1	89.3	76.8	0.795
Sokolow–Lyon criteria	72.7	62.1	2.28	99.5	4.42	0.506*
Cornell product criteria	78.5	61.8	0.39	99.9	0.77	0.568*
Cornell/strain index	58.0	62.6	6.90	96.9	12.3	0.517*
Framingham criteria	60.1	66.5	28.4	88.3	38.6	0.581*
Peguero–Lo Presti criteria	57.5	64.4	18.9	91.3	28.4	0.562*

LVH, left ventricular hypertrophy; PPV, positive predictive value; NPV, negative predictive value; AUC, area under the receiver operating characteristic curve.

*P < 0.05, compared with the AUC of CatBoost algorithm.

**Table 3:** Diagnostic performance of CatBoost predicting LVH in the validation sample among various subpopulations

	Sensitivity, %	Specificity, %	AUC
Age			
≥65 years, *n* = 4445	73.3	82.9	0.781
<65 years, *n* = 3958	63.3	93.7	0.785
Gender			
Male, *n* = 4625	68.1	90.1	0.791
Female, *n* = 3778	72.0	87.4	0.797
Hypertension			
Yes, *n* = 2896	77.3	80.4	0.789
No, *n* = 5507	63.3	92.0	0.776
Diabetes			
Yes, *n* = 1015	75.3	79.2	0.772
No, *n* = 7388	69.0	90.0	0.795
CAD			
Yes, *n* = 1361	79.8	78.7	0.793
No, *n* = 7042	67.2	90.4	0.788
Prior MI			
Yes, *n* = 450	79.8	79.2	0.795
No, *n* = 7953	69.9	88.9	0.794
Atrial fibrillation			
Yes, *n* = 724	83.1	83.5	0.833
No, *n* = 7679	68.4	89.4	0.789
COPD,			
Yes, *n* = 391	68.2	88.2	0.782
No, *n* = 8012	70.0	89.0	0.795

LVH, left ventricular hypertrophy; CAD, coronary artery disease; MI, myocardial infarction; COPD, chronic obstructive pulmonary disease; AUC, area under the receiver operating characteristic curve.

### Prognostic associations with left ventricular hypertrophy

**Figure 2:**
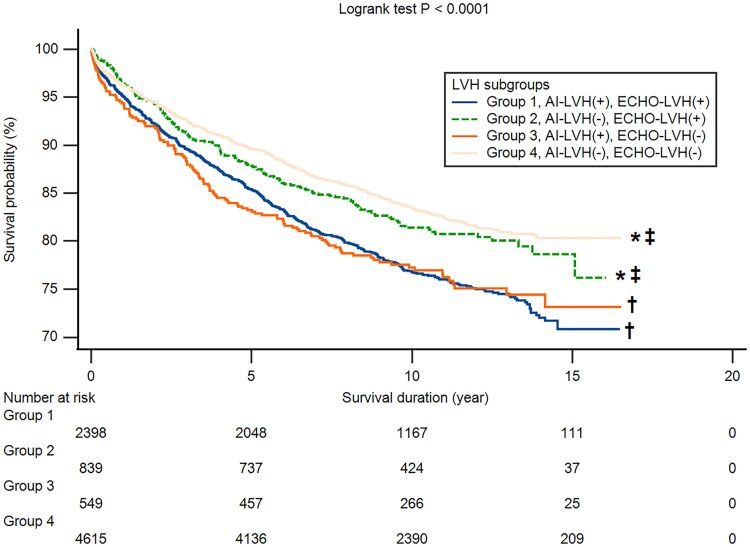
The Kaplan–Meier survival curve analyses of all-cause mortality stratified by the presence or absence of left ventricular hypertrophy, defined by echocardiography (ECHO-LVH) or artificial intelligence-interpreted electrocardiogram (AI-LVH). Group 1: both ECHO-LVH and AI-LVH, group 2: ECHO-LVH but not AI-LVH, group 3: AI-LVH but not ECHO-LVH, group 4: neither ECHO-LVH nor AI-LVH. *, ^†^, and ^‡^ indicated a log rank *P* < 0.05 for pairwise comparisons with groups 1, 2, and 3, respectively.

**Figure 3:**
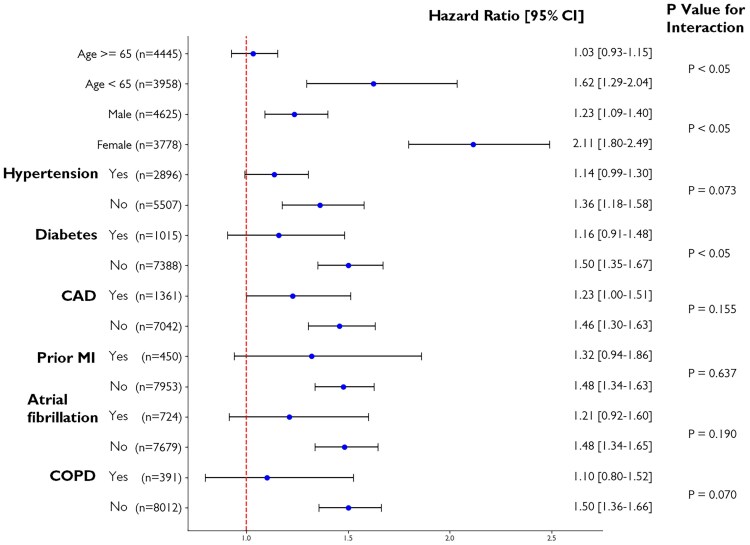
Subgroup analysis of AI-detected left ventricular hypertrophy in the prediction of all-cause mortality. The hazard ratio and 95% confidence interval across different subgroups were presented.

During a median follow-up duration of 10.1 years, there were 1655 deaths in the test group. While ECHO-LVH was significantly associated with total mortality [hazard ratio (HR) and 95% confidence intervals (CIs): 1.477, 1.340–1.628], all the conventional ECG-LVH criteria were also predictive of all-cause mortality except Peguero–Lo Presti (*Table 4*). After adjusting for age, gender, hypertension, diabetes, CAD, prior MI, AF, and chronic obstructive pulmonary disease, both CatBoost-defined LVH (HR: 1.387, 95% CI: 1.176–1.637) and ECHO-LVH (HR: 1.124, 95% CI: 1.019–1.239) remained significantly associated with mortality. Additionally, AI-LVH improved mortality prediction with a NRI of 3.46%, compared to 1.21% for ECHO-LVH. Kaplan–Meier survival curve analysis revealed that subjects with both echocardiography-defined and CatBoost-predicted LVH had the poorest survival outcomes (*Figure 2*). Notably, LVH identified solely by CatBoost was associated with a higher mortality risk compared to LVH diagnosed exclusively by echocardiography. In subgroup analysis, AI-LVH was consistently associated with an increased risk of all-cause mortality across various subpopulations (*Figure 3*). Notably, this elevated mortality risk was more pronounced in younger individuals compared to the elderly, in women compared to men, and in non-diabetic participants compared to those with diabetes (*P* for interaction < 0.05).

**Table 4:** Hazard ratios and 95% confidence intervals of echocardiography and electrocardiogram defined left ventricular hypertension for the prediction of mortality

Variable	Model 1 HR (95% CI)	*P* value	Model 2 HR (95% CI)	*P* value
Echocardiography	1.477 (1.340–1.628)	<0.001	1.124 (1.019–1.239)	0.020
CatBoost predicted	1.587 (1.309–1.924)	<0.001	1.387 (1.176–1.637)	<0.001
Sokolow–Lyon predicted	1.190 (1.038–1.365)	0.013	1.076 (0.974–1.188)	0.148
Cornell product predicted	1.301 (1.124–1.505)	<0.001	1.217 (0.826–1.794)	0.321
Cornell/strain index predicted	1.306 (1.185–1.439)	<0.001	1.249 (1.020–1.530)	0.032
Framingham criterion predicted	1.174 (1.062–1.298)	0.002	1.086 (0.937–1.259)	0.274
Peguero–Lo Presti criteria predicted	1.118 (0.974–1.282)	0.112	0.971 (0.862–1.092)	0.621

Model 1: non-adjusted; Model 2: adjusted for age, sex, hypertension, diabetes mellitus, coronary artery disease, prior myocardial infarction, atrial fibrillation, and chronic obstructive pulmonary disease.

### Feature selection

**Figure 4:**
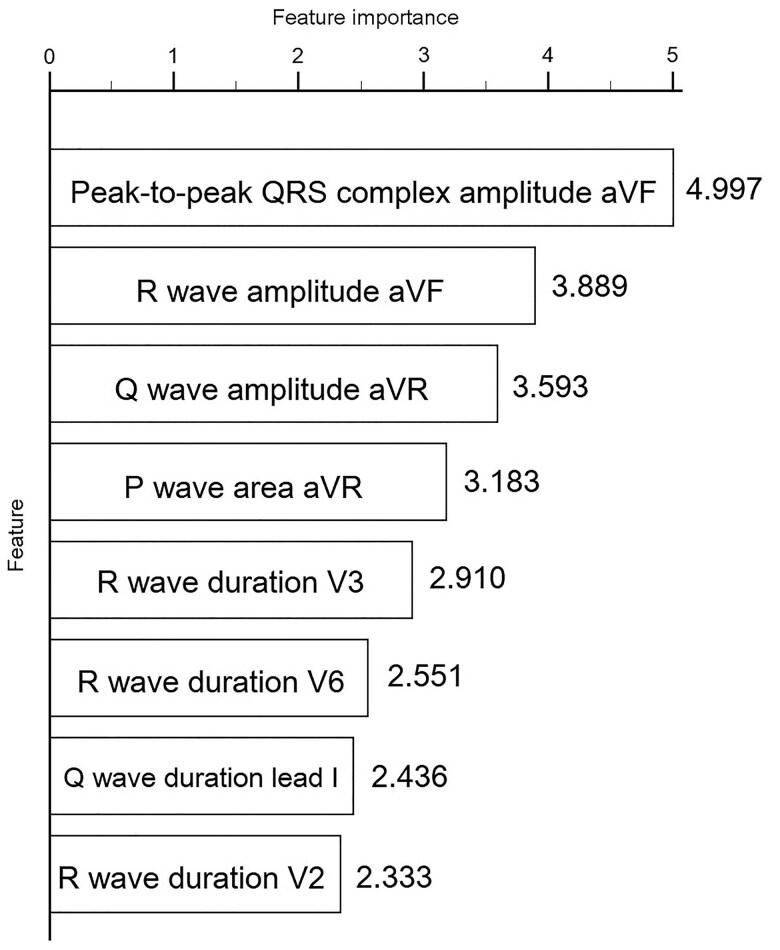
Feature selection by CatBoost model.

The importance of each feature was calculated using the feature selection in CatBoost analysis, and we plotted those with higher importance among the 527 ECG components (*Figure 4*), including peak-to-peak QRS complex amplitude of aVF, R-wave amplitude of aVF, Q-wave amplitude of aVR, P-wave area of aVR, R-wave duration of V3, R-wave duration of V6, R-wave duration of aVL, Q-wave duration of lead I, and R-wave duration of V2.

## Discussion

The present study demonstrated that conventional ECG-LVH criteria exhibited poor diagnostic accuracy due to low sensitivity in an Asian population. Nevertheless, ECG-predicted LVH was associated with long-term survival. The application of machine learning clearly improved the performance of ECG for LVH screening, which further disclosed that electrical remodelling might not only involve QRS but also P-wave morphology. In addition, LVH defined by a machine learning-based algorithm was superior to echocardiograms in the prediction of long-term mortality, further improving risk stratifications. The present study proposes the feasibility of using AI-interpreted ECG as a standard tool to screen for LVH.

### Electrocardiogram for the diagnosis of left ventricular hypertrophy

ECG-based screening for LVH represents a rapid and cost-effective tool that has been routinely applied to facilitate early intervention for cardiovascular diseases. Nowadays, at least 38 ECG-LVH criteria have been proposed, majority involving QRS morphology.^17^ However, the computer simulation demonstrated that diffuse or regional slowing in left ventricular conduction velocity could be the result of alterations in the sequence of ventricular activation, even though anatomical features, such as the mass and the shape of the left ventricle, were unchanged.^16,28^ Therefore, LVH would not necessarily be reflected by ECG criteria, which may be an explanation for the observed unsatisfactory diagnostic accuracy.^28,29^ Efficient screening for LVH using ECG has been challenging because of the low sensitivity and subsequent false-positive results.^28,29^ The AI approach could evaluate ECG characteristics more thoroughly and perform as a modern predictive model for LVH. In this study, the machine learning model outperformed traditional ECG criteria in LVH prediction, in terms of higher sensitivity and specificity, superior positive predictive value, and F1 scores.

Liu *et al*. developed several machine learning models, including Random Forest, Bayesian additive regression trees, and back-propagation neural networks, using a dataset of 952 patients (173 with LVH). These models were enhanced by ECG beat segmentation and focused on extracting R-peak and S-valley amplitudes from 12-lead ECG signals. They demonstrated improved accuracy and sensitivity in detecting LVH compared to traditional criteria, underscoring the potential of machine learning approaches in analysing ECG signals and advancing LVH diagnosis.^30^ In our study, we observed the superiority of machine learning models over traditional criteria. By leveraging a larger cohort and integrating additional ECG signal features, we significantly enhanced the model’s predictive capability. Moreover, the inclusion of prognostic data enabled survival analysis, highlighting the clinical utility of these models in predicting mortality. Khurshid *et al*. developed a deep learning model based on a convolutional neural network to estimate LVM from 12-lead ECGs and predict LVH, validated using cardiac MRI. Their model demonstrated superior accuracy in LVH detection and strong predictive power for cardiovascular risk.^31^ While we recognize that cardiac MRI is the current gold standard for assessing cardiac anatomy, the study used echocardiography due to its practical feasibility with a large dataset and its widespread applicability in routine clinical settings. Additionally, the improvements in sample size and the inclusion of extended follow-up data enhanced the robustness of our findings.

### Importance electrocardiography features in detecting left ventricular hypertrophy

QRS morphology is theoretically altered in a dilated or hypertrophic LV, which is the traditional ECG criteria basis. However, structural changes involving LVH might not be limited to LV *per se*. Left atrial dilatation is commonly observed in patients with LVH, which was viewed as an additional useful feature of ECG-LVH in a previous study.^32^ Garza-Salazar *et al*.^33^ demonstrated that negative P-wave deflection in V1 and right-side QRS morphology are associated with LVH. Although AI technology cannot explain the decision process, Kwon *et al*.^34^ showed that an AI algorithm concentrated on not only the QRS complex but also P-wave and T-wave morphology during a difficult task, based on heat map analysis. In our analysis, the CatBoost model demonstrated significant advancements in diagnosing LVH by incorporating nuanced features beyond traditional ECG criteria, such as P-wave morphology, QRS duration, and axis. Unlike conventional approaches that primarily rely on QRS voltage, CatBoost captured subtle electrical and vectorial characteristics of LVH. Notable findings included the directional significance of R-wave amplitude in lead aVF and Q wave amplitude in lead aVR, aligning with a 60–90° axis in the frontal plane, reflecting the spatial orientation of LV hypertrophy. Additionally, the R-wave duration in lead V3 was identified as a critical feature, consistent with the Peguero and Lo Presti criteria, further validating the model’s ability to detect LVH-associated electrical remodelling.

The inclusion of P-wave morphology is particularly significant, as it may reflect subtle changes associated with left atrial enlargement—a common consequence of LVH. Left atrial enlargement, often caused by prolonged pressure overload or volume changes, manifests as alterations in P-wave duration, amplitude, or morphology. Furthermore, ECG features such as R-wave characteristics in V2 and V3 likely reflect septal conduction. Strain patterns, including R waves in V6 and Q wave duration in lead I, were also identified as critical markers for diagnosing LVH.

### Left ventricular hypertrophy and long-term survival

Left ventricular hypertrophy detected by traditional ECG criteria has been linked to adverse cardiovascular events, and its regression has been shown to reduce associated risks, despite the low sensitivity of these criteria for detecting LVH.^5^ Therefore, early diagnosis and timely initiation of therapy could therefore improve clinical outcomes for affected patients. Evidence from prospective cohort studies suggested that ECG-LVH, defined by either the Cornell criteria or the Minnesota ECG Classification criteria, was associated with an increased incidence of heart failure and cardiovascular diseases.^29,35,36^ However, its prognostic value is inferior to imaging-based LVH diagnoses, as demonstrated by echocardiography in the Cardiovascular Health Study^29^ and cardiac magnetic resonance imaging in the Multi-Ethnic Study of Atherosclerosis.^36^ In this study, we also demonstrated that ECG-LVH diagnosed by conventional criteria was associated with long-term survival, albeit with prognostic performance inferior to ECHO-LVH. However, AI-predicted LVH from ECG outperformed ECHO-LVH in mortality prediction. Additionally, AI-predicted LVH (3.46%) offered significant added value by accurately reclassifying subjects into appropriate risk categories, providing a 2.25% improvement over ECHO-LVH (1.21%) when added to conventional risk factors.

### Clinical application and utility of artificial intelligence-predicted left ventricular hypertrophy

By outperforming both conventional ECG criteria and echocardiography in detecting LVH and predicting mortality, AI-LVH has the potential to revolutionize LVH diagnosis and risk stratification. This refined stratification could lead to improved clinical outcomes by facilitating timely management of LVH and its associated cardiovascular risks.

Moreover, the widespread accessibility of ECG and the integration of AI-LVH into routine clinical workflows may offer solutions for improving LVH detection, particularly in resource-limited settings where advanced imaging modalities like echocardiography or cardiac MRI may be unavailable. The superior prognostic value of AI-LVH also positioned it as a valuable tool not only for diagnosing LVH but also for ongoing risk monitoring, enabling personalized patient care and optimized treatment strategies. The study findings suggested that AI-LVH could bridge existing diagnostic gaps and enhance the overall management of patients with cardiovascular diseases.

#### Study limitations

These results should be interpreted in the context of certain limitations. First, we did not compare all available ECG criteria for LVH screening; however, the five criteria included in this study are among the most widely used and clinically relevant. Second, our study was conducted exclusively on a Taiwanese population from a single hospital, which may limit the generalizability of the study findings. However, the stratified analysis demonstrated consistent performance of the AI-based algorithm in detecting LVH across various subpopulations. Third, we employed the Teichholz method for LV mass estimation, appreciated for its simplicity, speed, and accessibility, making it well-suited for routine clinical use and resource-limited settings. Using M-mode echocardiography, it provides quick and reproducible measurements, enabling immediate clinical decision-making. However, its reliance on geometric assumptions, such as a symmetric, prolate LV shape, limits its accuracy in evaluating concentric vs. eccentric LVH and atypical ventricular geometries. While practical for routine applications, the Teichholz method may not be ideal for patients with complex LV shapes, where advanced imaging modalities like 3D echocardiography or cardiac MRI offer greater precision.

## Conclusion

The utilization of AI techniques for ECG algorithm training has overtaken conventional ECG criteria in the prediction of LVH. The feature selection method further suggested that P-wave morphology should be considered as a crucial component when performing ECG evaluations, and further work should also be conducted to compute new criteria based on AI models for bedside evaluation. While most of the ECG-based LVH cases were associated with long-term mortality, the prognostic value of AI-interpreted LVH was superior to ECHO-LVH. These results support the incorporation of AI technology into ECG interpretation to improve patient risk stratification.

## Supplementary Material

ztaf003_Supplementary_Data

## Data Availability

Access to de-identified data supporting this study requires a reasonable written request to the corresponding author.

## Lead author biography

Shih-Hsien Sung earned his MD and PhD from National Yang-Ming University, Taiwan, in 2001 and 2014, respectively. Since 2019, he has been a professor at National Yang Ming Chiao Tung University and an attending physician at Taipei Veterans General Hospital, where he completed internal medicine and cardiology training. He has pursued molecular biology in Japan, echocardiographic research in Hong Kong, and transcatheter valvular intervention in Germany. His research focuses on cardiovascular haemodynamics, heart failure, valvular heart disease, and pulmonary artery hypertension. Clinically, he specializes in heart failure care, transcatheter valvular and coronary interventions, and echocardiography.
