# Supplementary material for: Comparison of machine learning and conventional criteria in detecting left ventricular hypertrophy and prognosis with electrocardiography
Source: Eur Heart J Digit Health. 2025 Feb 11;6(2):252–60. doi: 10.1093/ehjdh/ztaf003 (PMC11914727; doi:10.1093/ehjdh/ztaf003)
Supplement: ztaf003_Supplementary_Data [file ztaf003_supplementary_data.docx]

S1 Table. A list of the ECG parameters analyzed by the Philips DXL-16 algorithm**.**

| **Parameter** | **Units or Value** | **Description** | **Type of variable** |
| --- | --- | --- | --- |
| **Morphology lead measurements** | | |  |
| **P AMP** | mV | P wave amplitude | Numeric |
| **P DUR** | msec | P wave duration | Numeric |
| **P AREA** | Ashman units  (40 msec x 0.1 mV) | P wave area for monophasic P waves or the area of the initial portion of a biphasic P wave | Numeric |
| **P’ AMP** | mV | P’ wave amplitude | Numeric |
| **P’ DUR** | msec | P' wave duration | Numeric |
| **P’ AREA** | Ashman units  (40 msec x 0.1 mV) | Area of the terminal portion of a biphasic P wave | Numeric |
| **Q AMP** | mV | Q wave amplitude | Numeric |
| **Q DUR** | msec | Q wave duration | Numeric |
| **R AMP** | mV | R wave amplitude | Numeric |
| **R DUR** | msec | R wave duration | Numeric |
| **S AMP** | mV | S wave amplitude | Numeric |
| **S DUR** | msec | S wave duration | Numeric |
| **R’AMP** | mV | R' wave amplitude | Numeric |
| **R’DUR** | msec | R' wave duration | Numeric |
| **S’AMP** | mV | S' wave amplitude | Numeric |
| **S’DUR** | msec | S' wave duration | Numeric |
| **V.A.T.** | msec | Ventricular Activation Time is the interval from the onset of the QRS complex to the latest positive peak in the complex, or the latest substantial notch on the latest peak (whichever is later) | Numeric |
| **QRS PPK** | mV | Peak-to-peak QRS complex amplitude | Numeric |
| **QRS DUR** | msec | QRS complex duration, measured from its onset to  the ST segment onset (J point) | Numeric |
| **QRS AREA** | Ashman units  (40 msec x 0.1 mV) | The area of the QRS complex | Numeric |
| **ST ON** | mV | Elevation or depression at the onset (J point) of the ST segment | Numeric |
| **ST MID** | mV | Elevation or depression at the midpoint of the ST segment | Numeric |
| **ST 80ms** | mV | Elevation or depression of the ST segment 80 ms after the end of the QRS complex (J point) | Numeric |
| **ST END** | mV | Elevation or depression at the end of the ST segment | Numeric |
| **ST DUR** | msec | ST segment duration | Numeric |
| **ST SLOPE** | degrees | ST segment slope. Slope is measured in degrees for 25 mm/sec, 1mV/cm scaling, and can range from -90 to +90 degrees. | Numeric |
| **ST SHAPE** | -, V, or ^ | The ST segment shape:  - = Straight  V = Concave upward  ^ = Concave downward | Categorical |
| **T AMP** | mV | T wave amplitude | Numeric |
| **T DUR** | msec | T wave duration | Numeric |
| **T AREA** | Ashman units  (40 msec x 0.1 mV) | T wave area for monophasic T waves or the area of the initial portion of a biphasic T wave | Numeric |
| **T’AMP** | mV | T' wave amplitude | Numeric |
| **T’DUR** | msec | T' wave duration | Numeric |
| **T’AREA** | Ashman units  (40 msec x 0.1 mV) | Area of the terminal portion of a biphasic T wave | Numeric |
| **PR INT** | msec | Interval from the onset of the P wave to the onset of the QRS complex | Numeric |
| **PR SEG** | msec | Interval from the end of the P wave to the onset of the QRS complex | Numeric |
| **QT INT** | msec | Interval from the onset of the QRS complex to the end of the T wave | Numeric |
| **Derived transverse QRS vector** | | |  |
| **Initial** | Vector angle in degrees  Vector magnitude in mV | The vector for the initial (first 40 msec) transverse QRS signal | Numeric |
| **Maximum** | Vector angle in degrees  Vector magnitude in mV | The maximum transverse QRS vector | Numeric |
| **Terminal** | Vector angle in degrees  Vector magnitude in mV | The vector from the terminal (last 40 msec) or last part of the transverse QRS signal | Numeric |
| **Rotation** | 100 to -100 | The direction of the vector rotation over the entire QRS complex   - A positive rotation value indicates a clockwise vector rotation - A negative rotation value indicates a counterclockwise vector rotation - A larger magnitude indicates a higher confidence in the rotation estimate | Numeric |
| **Frontal/Horizontal plane axis parameters** | | |  |
| **P** | Degrees or indeterminate | Mean P wave axis | Numeric |
| **I:40** | Degrees or indeterminate | Initial 40ms QRS complex axis | Numeric |
| **QRS** | Degrees or indeterminate | Mean QRS complex axis | Numeric |
| **T:40** | Degrees or indeterminate | Terminal 40 msec QRS complex axis | Numeric |
| **ST** | Degrees or indeterminate | Mean ST wave axis | Numeric |
| **T** | Degrees or indeterminate | Mean T wave axis | Numeric |
| **Global measurements** | | |  |
| **Mean Ventr Rate** | Beats per minute | Representative ventricular rate for the entire ECG | Numeric |
| **Mean PR Int** | msec | Representative PR interval for the entire ECG | Numeric |
| **Mean PR Seg** | msec | Representative PR segment for the entire ECG | Numeric |
| **Mean QRS Dur** | msec | Representative QRS duration for the entire ECG | Numeric |
| **Mean QT Int** | msec | Representative QT interval for the entire ECG | Numeric |
| **Mean QTc** | msec | Representative QT interval adjusted for heart rate | Numeric |
| **QT Dispersion** | msec | Difference between the longest and the shortest QT interval in the entire ECG | Numeric |

Abbreviations: msec=milliseconds, mV=millivolts

| Table S2. Diagnostic Performance of ECG LVH Criteria in the validation Sample (n=8403) | | | | | | |
| --- | --- | --- | --- | --- | --- | --- |
| LVH criteria | Sensitivity, % | Specificity, % | PPV, % | NPV, % | F1 score, % | AUC |
| XGBoost predicted | 79.6 | 82.8 | 72.3 | 85.4 | 75.6 | 0.689* |
| LightGBM predicted | 75.3 | 72.8 | 69.7 | 84.5 | 72.3 | 0.677* |
| RandomForest predicted | 73.4 | 76.5 | 68.6 | 84.2 | 71.2 | 0.643* |
| ExtraTrees predicted | 71.6 | 77.2 | 71.8 | 83.6 | 71.7 | 0.604* |
| *P<0.05, compared with CatBoost predicted  PPV= positive predictive value; NPV= negative predictive value, AUC= area under the Receiver Operating Characteristic curve | | | | | | |
